# Systematic laboratory approach to produce Mg-rich carbonates at low temperature†

**DOI:** 10.1039/d1ra06206a

**Published:** 2021-11-18

**Authors:** Zulfa Ali Al Disi, Nabil Zouari, Essam Attia, Mazen Al-Asali, Hamad Al Saad Al-Kuwari, Fadhil Sadooni, Maria Dittrich, Tomaso R. R. Bontognali

**Affiliations:** Environmental Sciences Program, Department of Biological & Environmental Sciences, College of Arts Sciences, Qatar University P. O. Box 2713 Doha Qatar zaldisi@qu.edu.qa; Environmental Science Centre, Qatar University P. O. Box 2713 Doha Qatar; Central Laboratory Unit, Qatar University P. O. Box 2713 Doha Qatar; Department of Physical and Environmental Sciences, University of Toronto Scarborough 1265 Military Trail Toronto M1C 1A4 Canada; Space Exploration Institute (SPACE-X) 68 Faubourg de l'Hopital 2000 Neuchatel Switzerland; Department of Environmental Sciences, University of Basel Klingelbergstrasse 27 4056 Basel Switzerland

## Abstract

Dolomite is a common Mg-rich carbonate in the geological record, but the mechanism of its formation remains unclear. At low temperature, the incorporation of magnesium ions into the carbonate minerals necessary to form dolomite is kinetically inhibited. Over the decades, several factors that possibly allow for overcoming this kinetic barrier have been proposed, and their effectiveness debated. Here, we present the results of a large number of laboratory precipitation experiments that have been designed to identify and compare the factors that promote the formation of Mg-rich carbonates. Under the tested conditions, the most interesting observations include: (1) from solutions that mimic evaporitic seawater, the maximum mol% of Mg incorporated in high Mg calcite is 35, (2) carbonates with a mol% of Mg above 40 were obtained exclusively in the presence of organic molecules, (3) no correlation was observed between the charge of the organic molecules and the incorporation of Mg, (4) the mode (*i.e.*, slow *vs.* fast mixing) used to add carbonate to the solution obtaining supersaturation has a significant impact on the forming mineral phase (aragonite *vs.* nesquehonite *vs.* high Mg calcite) and its Mg content. These findings allow for a more informed evaluation of the existing models for dolomite formation, which are based on the study of natural environments and ancient sedimentary sequences.

## Abstract 2

Carbonates with a mol% of Mg above 40 were obtained exclusively in the presence of organic molecules. The mode used to add carbonate to the solution obtaining supersaturation has a significant impact on the forming mineral phase.

## Graphical abstract

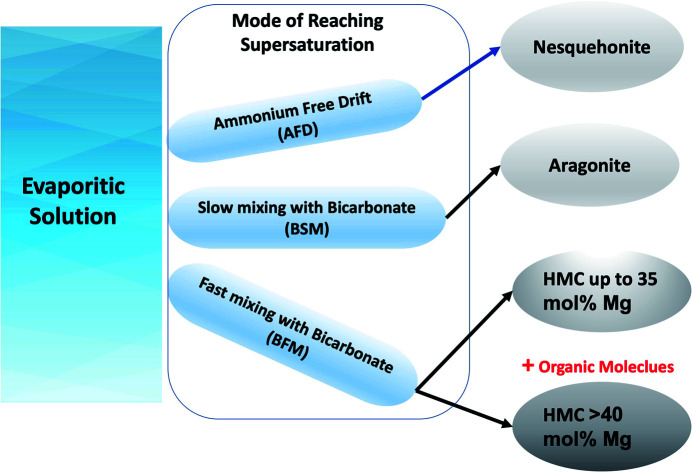

## 1 Introduction

Dolomite – MgCa(CO_3_)_2_ – is a common carbonate mineral in ancient sedimentary sequences. Despite the fact that seawater is supersaturated with respect to dolomite, its formation as a primary or early diagenetic precipitate is very rare in modern environments.^1,2^ Laboratory experiments corroborate this observation showing that at temperatures lower than 60–80 °C, the incorporation of Mg into anhydrous carbonate minerals is kinetically inhibited.^3–6^ Because the thick strata of dolomite present in ancient sedimentary sequences are practically impossible to explain as a result of a high temperature metamorphic/replacement process (*e.g.*, through a replacement process whereby Mg is transported by diffusion),^7,8^ factors that under some circumstances allow for overcoming such a kinetic barrier leading to the formation of dolomite as a primary or early diagenetic mineral must exist. The identification of such factors is at the center of a debate that is often referred to as “the dolomite problem”.^2,4,9,10^

During the last two centuries, many models for dolomite formation have been proposed, without providing an unanimously accepted solution to the problem. In the last decades, the hypothesis that gained more momentum than others posits that microbes may play a mediating role in the mineralization process (*e.g.* ref. 4 and 11–17). Extracellular polymeric substances (EPS) that microorganisms secrete from their cells to obtain ecological advantages – including both large polymers and low molecular weight organic matter^18,19^ – have been shown to be of a particular importance for the process. Besides providing nucleation sites, such organic molecules may promote the dehydration of Mg, which is a recognized kinetic barrier for the formation of Mg-rich carbonates.^2,5,15,20–22^ It is known that EPS are secreted by microorganisms into their microenvironment as response to ecological stress.^18,23^ EPS are usually produced in huge amounts as a protective strategy to overcome extreme environmental variations (*e.g.*, evaporation and subsequent desiccation and increase in salinity).^24^

Sabkhas – the Arabic term for flat saline mud areas – are modern dolomite-forming environments where such stresses are exerted on microbial populations.^25^ Sabkhas occur in regions characterized by extreme climatic and environmental conditions, such as high temperature, salinity, light intensity, *etc.*^26,27^ Since long, sabkhas have been studied as modern analogues for interpreting some ancient dolomite-rich sedimentary sequences, and several models for dolomite formation are based on the study of these depositional settings.^28^ More recent studies indicate that not only evaporation, but also microbial processes may play a key role in these evaporitic environments.^2,29,30^ It has been suggested that the strong evaporation causing consequently high salinity and increased supersaturation, combined with microbial EPS produced under extreme ecological stress, may be linked to the formation of dolomite at ambient temperature. Supersaturated pore-waters, as well as high alkalinity and suitable redox conditions sustained by microbial respiration in buried microbial mats,^2^ may create the chemical conditions required for low temperature dolomite formation. Recent works have focused on the community composition of microbial mats (with top layers comprised mostly of cyanobacteria and anoxygenic phototrophs) that promote the formation of dolomite in a sabkha environment, revealing that anoxygenic phototrophic microbes are particularly important for the mineralization process.^10,27^ Specifically, a cyclic shift in microbial community between cyanobacteria and anoxygenic phototrophs results in the production of EPS with an increased concentration of carboxylic functional groups, which in turn favors dolomite formation.

Considering the almost ubiquitous presence of microbes and EPS in modern environments where dolomite is not forming, it can be deduced that only EPS with a specific composition play a catalytic role (*e.g.* ref. 15 and 27), or that EPS need to co-occur with other factors. The identification of co-occurring factors essential for dolomite formation cannot be easily inferred by reviewing and comparing the results of published studies, which are often conducted using different methodologies. For this reason, we have conducted laboratory precipitation experiments with the goal of systematically comparing which minerals form under various conditions, using different methods to reach supersaturation. To make the results of the experiment as relevant as possible for studies of natural environments, we performed experiments with artificial solutions that mimic the pore waters composition of the Dohat Faishakh sabkha in Qatar^28,30^ (see Map in ESI†). This sabkha has been the focus of previous studies, which led to the isolation of microbes capable of precipitating Mg-rich carbonates in the laboratory^14^ and the characterization of their EPS.^15^ Because the method used in laboratory experiments to reach supersaturation with respect to carbonate minerals may affect the kinetic of the reaction resulting in the formation of different phases^31^ we have used and compared three methods: ammonium free-drift (AFD), slow mixing with sodium bicarbonate method (BSM) and fast mixing with sodium bicarbonate method (BFM). To simulate EPS, we performed experiments adding different organic molecules that occur in natural EPS and provide nucleation surfaces and charged functional groups.^32^ We selected molecules characterized by different charges (*i.e.*, negative, positive, and neutral). This approach allowed us to test the hypothesis^5,33–35^ that negatively charged carboxyl groups are essential to promote Mg dehydration and consequent incorporation in Mg-rich carbonates.

## 2 Material and methods

### 2.1 Precipitation solutions

An artificial solution was prepared based on the results of ICP-mass analysis of some natural pore waters of a sabkha sediment (*i.e.*, the Dohat Faishakh sabkha) in which dolomite is currently forming^28,30^ (see also Fig. S1 and Table S1 in ESI†). The composition was (mM): MgCl_2_ 379, CaCl_2_ 25, NaCl 4053, KCl 67, NaSO_4_ 100, SrCl_2_ 0.3 and LiCl 0.1. For each experimental setup, different types of amino acid (w/v 0.1%) and/or xanthan (w/v 0.1%) were added. All solutions were prepared using Milli-Q water and sterilized by filtration through a 0.2 μm pore size membranes. Parallel control experiments were performed in the absence of organic molecules. As an additional control, experiments were also conducted with natural pore water from the Dohat Faishakh sabkha (labelled SW4), which was sterilized by filtration. The measured concentrations of the major ions of SW4 were similar to those of the artificial solution. Visual checks were regularly performed to exclude contamination and microbial growth in the vials. All experiments were carried out-at least-in duplicates. The saturation indices of the artificial solution at various pH were calculated as log *Q*/*K* using SpecE8-The Geochemist's Workbench (GWB2021)-Community Edition.

### 2.2 Organic compounds

Xanthan gum (Xan) is an extracellular polysaccharide produced by *Xanthomonas campestris* bacterium. Xanthan is composed of repeating units of mannose, glucose, glucuronic acid and pyruvic acid.^36^ Xanthan forms homogeneous aqueous dispersions and increases viscosity.^37,38^ With respect to artificially synthesized organic compounds, xanthan produced in different facilities or in different times may show slight compositional variabilities. Moreover, not only xanthan composition but also its use at different concentrations, which in turn affect viscosity of the precipitation environment, may impact crystal morphology and mineralogy.^37,38^

Six amino acids providing different functional groups were selected and used in this study. l-Glutamine (Gln) (C_5_H_10_N_2_O_3_) is polar with a side chain containing two amino groups. l-Glutamic acid (Glu) (C_5_H_9_NO_4_) and l-aspartic acid (Asp) (C_4_H_7_NO_4_) are acidic, polar, containing two carboxyl groups. Phenylalanine (Phe) (C_9_H_11_NO_2_) and alanine (Ala) (C_3_H_7_NO_2_) are non-polar. Arginine (Arg) (C_6_H_14_N_4_O_2_) is a basic amino acid (see Table S2 in ESI†). Stock solutions of amino acids were prepared and sterilized by filtration through a hydrophilic polyethersulfone (PES) filter membrane with a 0.22 micron pore size to remove biological contaminants, including bacteria. The xanthan stock solutions were sterilized and thus homogenized by autoclaving at 121 °C for 20 min.

The approach of using “single” amino acids with respect to xanthan or natural biofilms allows for evaluating the influence of specific functional groups for mineral precipitation. On the other hand, it fails to mimic the complexity of natural biofilms, in which a large variety of organic molecules with various functional groups co-occur at the mineral nucleation site.

### 2.3 Mineral precipitation experiments

#### 2.3.1 Ammonium free-drift method (AFD)

Precipitation experiments were performed using the ammonium carbonite (NH_4_)_2_CO_3_ free drift method.^39^ The Petri dishes containing combinations of precipitation solutions were placed in a sealed desiccator pre-sterilized using alcohol. Each Petri dish contained a total volume of 20 mL of the precipitation solution and was incubated at room temperature (*i.e.*, about 25 °C) in the 25 cm-diameter desiccator filled with 20 grams of ammonium carbonates. The decomposition of ammonium carbonate produces NH_3_, CO_2_ and H_2_O. Consequently, the dissolution of NH_3_ into the experimental solutions causes the increase of pH, while the CO_2_ dissolution provides CO_3_^2−^, which acts as a source for the precipitation of carbonate minerals. The precipitates were recovered after an incubation time ranging from two weeks to one month.

#### 2.3.2 Precipitation experiments by slow mixing with bicarbonate (BSM)

For the bicarbonate slow mixing method (BSM), 200 mL of precipitation solutions were placed in 1000 mL flasks. The precipitation solutions were initially flushed with CO_2_ for 5 h to reach a pH of 5.0 and then flushed with N_2_ for almost 3 h to reach a pH of 7.0. Then, a mini pump with variable flow set at 0.05 mL min^−1^ was used to slowly inject the 50 mL sodium bicarbonate (NaHCO_3_) solution to obtain a final sodium bicarbonate concentration of 100 mM. The N_2_ flushing was maintained for the next 24 h. After 24 h, precipitates were observed in all the experiments. The solutions and contained precipitates were rigorously mixed and then divided into four batches of sterile falcon tubes (25 mL each). The precipitates from the first batch (24 h batch) was recovered as described in Section 2.3.4, while the remaining batches were incubated at 30 °C for extended incubation periods of 1–3 months as specified in the results.

#### 2.3.3 Precipitation experiments by fast mixing with sodium bicarbonate (BFM)

The bicarbonate fast mixing experiments (BFM) were performed in 100 mL bottles containing 45 mL of precipitation solution. The solutions were bubbled with N_2_ using syringes. 5 mL of sodium bicarbonate solutions were added rapidly (about 5 seconds) to obtain a total volume of 50 mL and a final NaHCO_3_ concentration of 100 mM. Then, the bottles were incubated at 30 °C in an incubator during 24 h or 1–3 months before recovering the minerals.

#### 2.3.4 Scanning electron microscopy (SEM) and energy-dispersive X-ray (EDS) spectroscopy and X-ray diffraction (XRD) analysis

At the end of the incubation periods, the precipitated minerals were recovered by centrifugation at 5000*g* for 15 min. The minerals were washed three times with distilled water to remove extra salts and then air dried at 40 °C to be analyzed by SEM/EDS and XRD. Such washing procedure may have caused the dissolution not only of the salts but also of a minor fraction of other minerals.

SEM images were obtained using Nova Nano Scanning Electron Microscope equipped with Bruker EDS Detector with 5 nm resolution and a magnification of 2 000 00×. The EDS analyses were performed following the “ASTM standard method E1508-12a”.^40^

To determine their mineralogical composition, the recovered precipitates in form of dried powder were subjected to a discrete X-ray analysis. The bulk mineralogical composition was determined using a PANalytical-multipurpose Empyrean X-ray diffractometer. The analysis of XRD spectra was performed using the MATCH! Software, Version 3.11.2.188, CRYSTAL IMPACT, Kreuzherrenstr. 102, 53227 Bonn, Germany. The Mg mol% of carbonate minerals were calculated according to the position of the (*d*_104_) peak in the XRD pattern using the formula of Goldsmith *et al.*, (1961).^41^ No quartz was added as internal standard to the bulk precipitate. Nevertheless, most of the samples contained minor amounts of halite, which forms when the bulk mineral phases are separated from the artificial solution and dried. The halite (200) was therefore used to correct for minor shifts in the position of the *d*_(104)_ reflection of our synthetic Mg calcite.

For practical reasons (*e.g.*, use of Petri dishes *vs.* flasks and flasks connected to a gas flushing system), the volume of the precipitation solutions, the incubation-time, and the incubation temperature, varied depending on the used method. For details, see the paragraph above, the “Results and discussion” section, and Table 1. Maintaining sterility avoiding microbial contamination was more difficult with the AFD method with respect to the BSM and BFM methods. For this reason, the incubation times of the experiments conducted with the AFD method were kept shorter (*i.e.*, 2 weeks to 1 month *vs.* 1 to 3 months). However, one duplicate set of the experiments performed with the AFD method was transferred to centrifuge tubes and kept for a longer incubation period (*i.e.*, more than 3 months). No changes in the bulk mineral phases were observed, suggesting that the difference in incubation time does not represent a major bias for the comparison of the results obtained with the 3 different methods.

**Table 1:** Minerals formed during laboratory precipitation experiments in the presence of organic molecules using different methods

No.	Exp.	Ammonia free-drift (AFD) at 25 °C and 1 month incubation		Slow mixing conditions (BSM) at 30 °C and 1–3 months incubation		Fast mixing conditions (BFM) at 30 °C and 1–3 months incubation			
		Ending pH	Ending precip.	Ending pH	Ending precip.	Ending pH	Yield of ending precipitates		Mol% Mg
1	Cont.1	9.85 ± 0.32	Nesquehonite/minor amounts of aragonite & Mg-calcite	8.16 ± 0.21	Aragonite/miner amounts of calcite & huntite/hydromagnesite	8.10 ± 0.13	Aragonite (63%)	HMC (37%)	33.7 ± 0.9
2	Cont.2 (SW4)	9.67 ± 0.47		8.19 ± 0.11		8.01 ± 0.25	Aragonite (76%)	HMC (33%)	34.5 ± 1.2
3	Xan	9.73 ± 0.54		8.28 ± 0.07		7.88 ± 0.16	Aragonite (15%)	HMC (85%)	37.3 ± 2.3
4	Glu	9.48 ± 0.21		8.01 ± 0.01		7.80 ± 0.1	Aragonite (87%)	HMC (13%)	37.5 ± 1.2
5	Glu–Xan	9.50 ± 0.11		8.21 ± 0.13		7.94 ± 0.1	Aragonite (96%)	HMC (4%)	33.0 ± 2.3
6	Gln	9.88 ± 0.34		8.31 ± 0.07		8.40 ± 0.08	Aragonite (100%)	—	—
7	Gln–Xan	9.72 ± 0.17		8.40 ± 0.08		8.23 ± 0.11	Aragonite (7%)	HMC (93%)	38.9 ± 1.1
8	ASP	9.41 ± 0.04		8.27 ± 0.08		7.84 ± 0.11	Aragonite (87%)	HMC (13%)	36.2 ± 1.3
9	ASP–Xan	9.39 ± 0.26		8.20 ± 0.13		7.72 ± 0.19	Aragonite (94%)	HMC (6%)	31.28 ± 0.1
10	Phe	9.68 ± 0.09		8.33 ± 0.04		8.26 ± 0.24	Aragonite (18%)	HMC (82%)	42.19 ± 1.9
11	Phe–Xan	9.54 ± 0.52		8.37 ± 0.05		8.23 ± 0.17	Aragonite (6%)	HMC (94%)	40.2 ± 2.7
12	Ala	9.85 ± 0.37		8.33 ± 0.06		8.24 ± 0.02	Aragonite (100%)	—	—
13	Ala–Xan	9.82 ± 0.14		8.26 ± 0.07		7.99 ± 0.21	Aragonite (10%)	HMC (90%)	35.65 ± 0.3
14	Arg	9.98 ± 0.12		8.41 ± 0.08		8.25 ± 0.03	Aragonite (17%)	HMC (83%)	38.42 ± 0.8
15	Arg–Xan	9.95 ± 0.08		8.26 ± 0.06		8.22 ± 0.04	Aragonite (19%)	HMC (81%)	41.68 ± 0.5

## 3 Results and discussion

### 3.1 pH evolution at different mineral formation conditions and corresponding saturation indices

The pH was monitored in all experimental conditions using a bench pH meter (*i.e.*, pHenomenal IS 2100L). For the precipitation experiments performed using ADFM, the pH was adjusted to 7.00 at the beginning of the precipitation experiments. The pH evolved to 9.30 within 15 min and remained almost at same level until the end of the incubation period. For the BSM and BFM precipitation experiments, the reactant NaHCO_3_ was added to the precipitation solutions once the pH reached 7.00 by N_2_ bubbling. The pH increased to a range between 8.00 and 8.41 at the end of BSM incubation period. With the BFM method, final pH ranged from 7.72 to 8.25. The increase in solution's pH observed at the end of the experiments conducted with both the BFM and BSM methods (*i.e.*, slow and fast mixing of bicarbonate) is due to the bubbling of N_2_, which results in degassing of CO_2_. The significant increase in pH observed at the end of the experiments performed with the ammonium free-drift method (AFD) is due to the decomposition of (NH_4_)_2_CO_3_. The NH_3_ gas rapidly dissolves in solution leading to an increase in pH.

The calculated saturation indices show that the solutions were saturated with respect to dolomite, aragonite and calcite at pH 7. Upon the increase of pH to 8, the solutions become additionally saturated with respect to monohydrocalcite and hydromagnesite. At pH of 9 and above, the saturation index of nesquehonite is reached (see Table S3 in ESI†). Information on main ions activity and cations to anions ratios are reported in Tables S4 and S5.†

### 3.2 SEM/EDS and XRD analysis

**Fig. 1:**
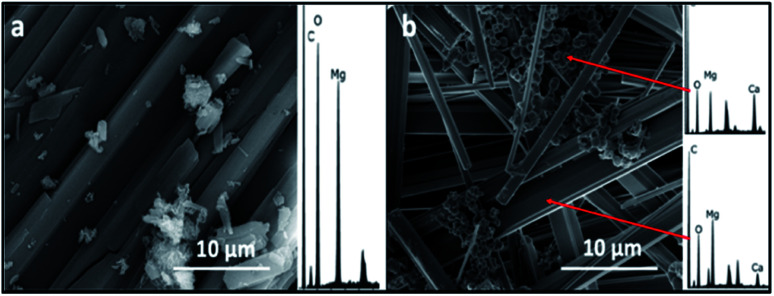
Representative SEM/EDS images and spectra of crystals recovered from precipitation experiments performed using ammonium free drift method, (a) control showing nesquehonite crystals, (b) spherical Mg calcite and columnar nesquehonite crystals formed in the presence of phenylalanine.

The SEM/EDS analysis of the minerals obtained at the different conditions, revealed high variations in the morphology and the elemental composition of the recovered minerals. In the experiments performed using AFD, the majority (>85%) of crystals formed in the presence/absence of amino acids and/or xanthan were columnar shaped magnesium carbonates (identified with XRD analysis as nesquehonite – (Mg(HCO_3_)(OH)·2(H_2_O))) besides minor amounts of spherical-shaped Mg–calcium carbonate crystals with variable Mg content (Fig. 1).

**Fig. 2:**
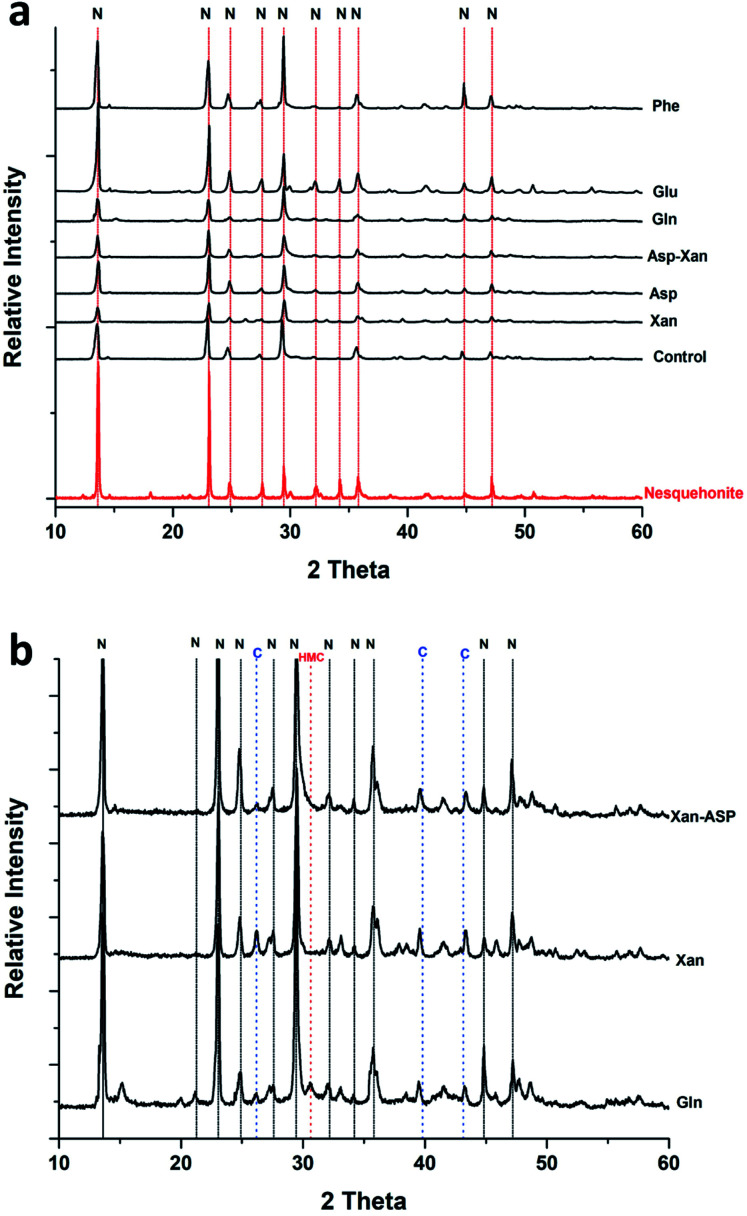
(a) Representative XRD patterns of minerals obtained from the precipitation experiments performed using the ammonium free-drift method. (b) Close up of X-ray diffraction patterns showing minor phases. N: nesquehonite, C: calcite, HMC: high magnesium calcite.

XRD analysis of the minerals formed using the AFD method (Table 1 and Fig. 2) revealed that nesquehonite is the main mineral phase together with minor (<10%) amounts of calcite and Mg-calcite.

**Fig. 3:**
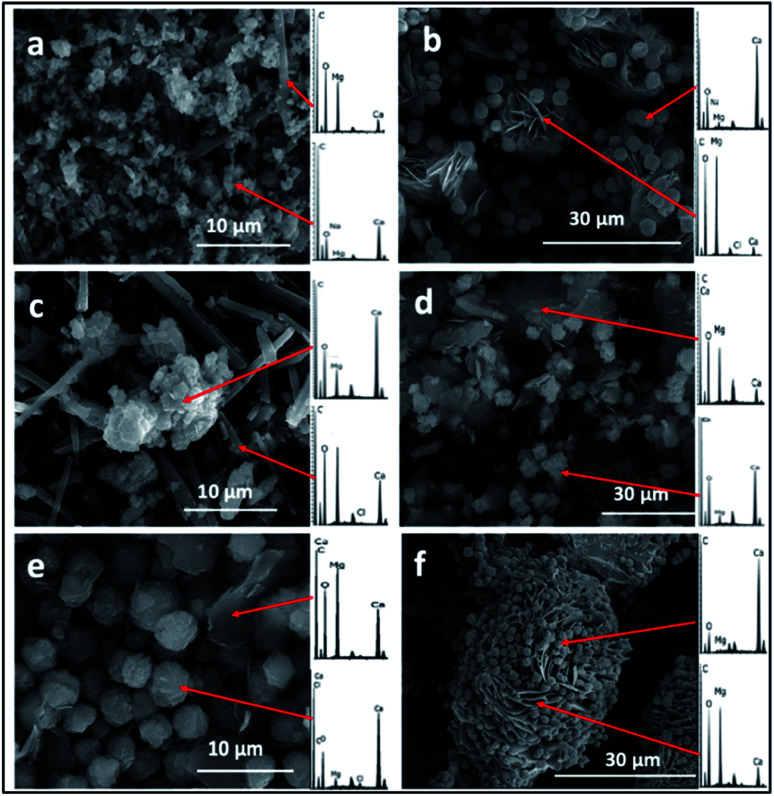
Representative SEM/EDS images and spectra of crystals recovered from the precipitation experiments performed using the BSM method, (a) crystals formed in a control experiment without organic molecules in 24 h, (b) crystals formed in a control experiment without organic molecules in 2 months, (c) crystals formed in the presence of aspartic acid in one month, (d) crystals formed in the presence of aspartic acid in 3 months, (e) crystals formed in the presence of xanthan in one month, (f) crystals formed in the presence of glutamine and xanthan in 3 months.

With the BSM method, the minerals recovered after 24 h were identified by SEM/EDS as calcium carbonate and Mg–calcium aggregates with minor amounts of columnar nesquehonite crystals. However, the SEM/EDS of the minerals recovered after longer incubation periods (1–3 months or more) showed growing semi-rounded shaped calcium carbonate crystals, but with lower amounts of incorporated magnesium. Moreover, the nesquehonite morphology transformed into a rosette aggregate (huntite/hydromagnesite), as illustrated in (Fig. 3).

**Fig. 4:**
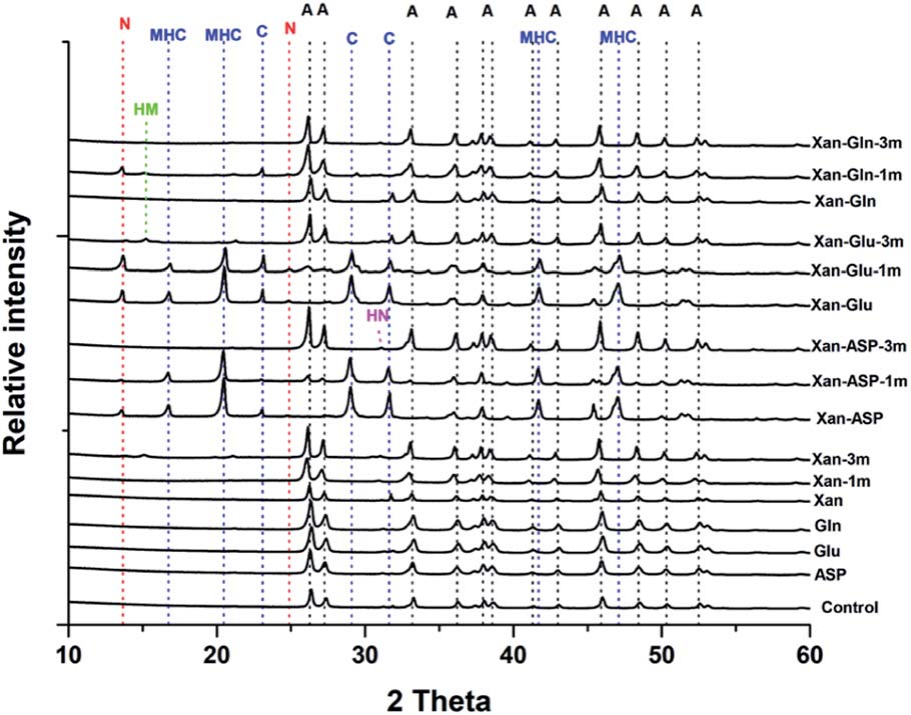
Representative XRD patterns of minerals recovered from the precipitation experiments performed with the BSM method. A: aragonite, MHC: monohydrocalcite, C: calcite, N: nesquehonite, HN: huntite, HM: hydromagnesite.

The XRD analysis of the minerals that formed in the precipitation experiments performed with the BSM method confirmed that aragonite is the main forming-phase in the presence of xanthan, of all the individually tested amino acids, and with the combination of xanthan and glutamine. Instead, monohydrocalcite/calcite were formed in the presence of glutamic acid or aspartic acid combined with xanthan (Table 1 and Fig. 4). This observation is consistent with the results of a recent study on microbially induced formation of monohydrocalcite, showing that glutamic and aspartic acids are preferentially adsorbed and mixed in the monohydrocalcite crystal lattice, and may have a role for its formation.^42^ In our experiments, the metastable monohydrocalcite phase transformed to aragonite after longer incubation periods (3 months and above) (ESI Fig. S2†). Such a transformation has been reported in previous studies, suggesting that monohydrocalcite that nucleates during simultaneous dissolution of Mg-bearing, amorphous calcium carbonate may be an important intermediate for aragonite and calcite that form at high Mg/Ca ratios.^43,44^ The minor amounts of nesquehonite transformed into huntite/hydromagnesite (Table 1, Fig. 4 and ESI Fig. S2†)

**Fig. 5:**
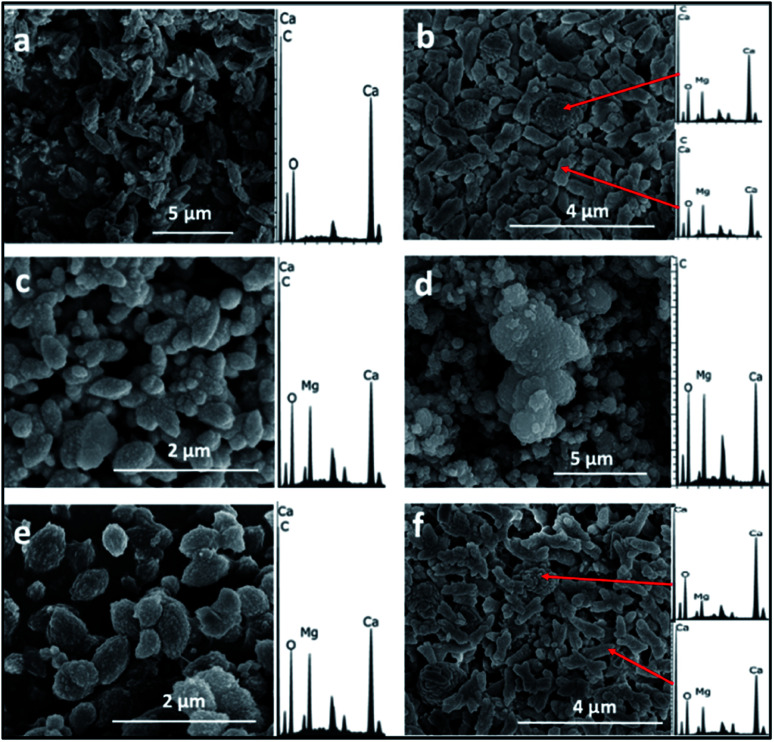
SEM/EDS of crystals recovered from precipitation experiments performed with the BFM method. (a) Control without organic molecules (b) experiment with xanthan, (c) with phenylalanine, (d) with phenylalanine and xanthan (e) with glutamine and xanthan, and (f) with alanine and xanthan.

The majority (>90%) of crystals formed with the BFM method in the control (*i.e.*, without organic molecules) or using amino acids were oval shaped with an average length of almost 1 μm. Mg-calcite crystals with various mol% Mg were observed in the precipitates that formed in the presence of organic molecules (Fig. 5). Crystals formed in the presence of xanthan have two different morphologies: oval-shaped (Fig. 5B and F) with higher content of Mg and aggregates forming semi-spherical shapes with lower Mg content. No major changes were observed in the morphology and composition of the minerals recovered after longer incubation periods (of 1–3 months).

**Fig. 6:**
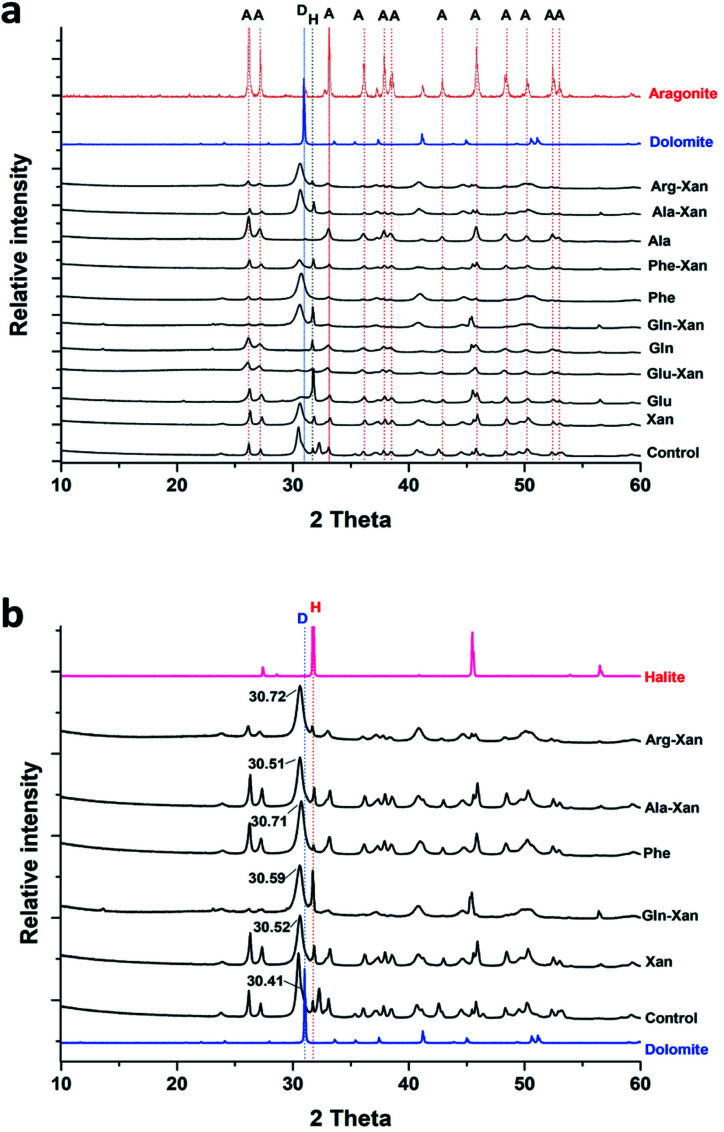
(a) Representative XRD patterns of minerals recovered from the precipitation experiments performed with the BFM method, (b) XRD patterns of the recovered HMC minerals showing the 2-theta values of the main peaks. A: aragonite, D: dolomite, H: halite.

Interestingly, with the BFM method high magnesium calcites (HMC) phases with variable mol% of Mg were obtained (Table 1 and Fig. 6). In the control samples, aragonite and HMC with a mol% up to 35 were formed. Aragonite was the mineral formed in presence of glutamine and alanine in absence of xanthan. In presence of xanthan, aragonite and HMC were formed with or without amino acids. The amounts of aragonite and HMC in each mixture were semi-quantitatively assessed using MATCH software. The highest amounts of HMC (81–94%) were obtained in the presence of phenylaniline, phenylaniline–xanthan, glutamine–xanthan, alanine–xanthan, arginine, and arginine–xanthan. The highest mol% Mg (38.9 ± 1.1 to 42.19 ± 1.9) was obtained in the presence of phenylaniline, phenylaniline–xanthan, glutamine–xanthan, and arginine.

The results of the control precipitation-experiments performed with natural pore waters (*i.e.*, Cont. 2–SW4) are broadly similar to those performed using the artificial solution (see Table 1).

Anhydrous Mg-carbonates with a mol% Mg higher than 40 – a phase that is considered as a possible precursor to ordered dolomite, referred in the literature to as protodolomite, non-ordered dolomite, Ca-dolomite, or very high Mg calcite^2,45^ – has been detected only in some of the experiments conducted with the bicarbonate fast mixing method (BFM) and in the presence of organic molecules (Table 1). Most of the tested experimental conditions only led to the formation of aragonite, calcite, and hydrous Mg-rich phases (*i.e.*, nesquehonite and hydromagnesite). These results are consistent with previous works, indicating that (1) incorporation of Mg into anhydrous carbonate minerals is kinetically inhibited at low temperature (*e.g.* ref. 46), (2) that aragonite and not calcite is forming from solutions with a high Mg/Ca ratio (*e.g.* ref. 47, 48 and 54) (3) that rapid rise in pH promotes the formation of nesquehonite,^49^ and (4) that the presence of organic molecules – produced by microbes in most natural environments – promote the incorporation of Mg into the carbonate mineral (*e.g.* ref. 5, 10 and 50). It is worth noting that our experiments were conducted in the absence of any mineral seed, which are also known to influence the nucleation of Mg-rich carbonates.^51–53^ In particular, a recent study has shown that the presence of seeds comprised of negatively charged clay minerals promote the incorporation of Mg into the carbonate mineral.^34^ Besides confirming the results of the abovementioned previous studies, our experiments provided novel insight on the factors influencing the incorporation of Mg into carbonate minerals at low temperature, which we discuss in more detail below.

Having conditions that promote the dehydration of Mg shells has often been described as essential in many microbial models for dolomite formation (*e.g.* ref. 2). Functional groups present in EPS have been often proposed as promoting dehydration due to their negatively charged functional groups.^33^ In the range of pH at which the BSM and BFM precipitation experiments were performed (*i.e.*, 7 to 8.5), the carboxyl groups of all the tested amino acids were deprotonated, and their amine groups were protonated (Table S2†). The total charge of the acidic and basic amino acids was determined by the charge of their side chain (see Table S2†). The results of our experiments do not point to any significant correlation between specific groups of amino acids and the incorporation of Mg into the carbonate mineral. For example, the presence of Glu and Asp – that under our experimental conditions had a negative charge – resulted in the formation of HMC with a mol% Mg that is not higher than that of the HMC formed in the presence of positively charged (*i.e.*, Arg) or neutrally charged (*i.e.*, Ala, Phe, Gln) amino acids (Table 1). In the case of the experiments conducted with the AFD method, pH values up to 9.9 were reached. Therefore, also the amine groups (expect that of arginine) were deprotonated (Table S2†). However, only minor amounts of HMC were detected. It could be the case that under the conditions mimicked by our experiments, the organic matrices tested exhibited higher affinity for Ca^2+^ than for Mg^2+^ ions. Additional experiments considering also binding site concentrations and competitive interactions between Mg^2+^ and Ca^2+^ for the available ligands, and at higher pH ranges, would help in concluding further on this matter.

Xu *et al.*, (2013)^48^-conducted precipitation experiments at low temperature in the absence of water and showed Mg hydration is probably not the only kinetic barrier preventing the formation of Mg carbonates. They proposed a more intrinsic inhibitory effect that may be due to lattice restraints on the spatial distribution of CO_3_ groups in the MgCO_3_ crystals. Without being able to provide a molecular scale model elucidating the role of organic molecules for the nucleation process, we concur with the hypothesis of Xu *et al.,*^48^ and suggest that kinetic barriers other than dehydration, which are more easily overcome in the presence of organic molecules^50^ but independently from their charge, probably exist.

Another interesting outcome of our experiments is the demonstration that the mode of carbonate supply (*i.e.*, not only the total inorganic carbon present in solution, but also the time at which supersaturation is reached), has a significant impact on the forming mineral phase (*i.e.*, aragonite *vs.* hydromagnesite *vs.* nesquehonite *vs.* high Mg calcite) and its Mg content. Indeed, at equal Mg^2+^ : Ca^2+^ ratios, the formation of high Mg calcite is favored by a rapid addition and mixing of carbonate ions (*i.e.*, experiments conducted with the BFM method). The concept that high alkalinity deriving from both physicochemical (*e.g.*, evaporation) and biological processes (*e.g.*, microbial metabolisms) may favor dolomite formation is not new (*e.g.* ref. 2, 55 and 56). However, despite several modern dolomite-forming environments are characterized by an alkalinity that surpasses that of average seawater (*e.g.* ref. 11, 27 and 50) there is evidence indicating that alkalinity is not the ultimate factor controlling whether Mg-carbonates *vs.* other carbonate phases will form at low temperature (*e.g.* ref. 57 and 58). Nevertheless, it is important to note that the debate about the importance of high alkalinity for the formation of Mg-rich carbonates mostly focuses on the concentration in solution of carbonate ions in solution and not on the mode and velocity/rate they are brought to the site of mineral nucleation and growth. Based on the results of this study, we propose that the mode of carbonate supply may play an important and, as yet, limitedly investigated role for the formation of Mg-rich carbonates.

It is possible that the formation of Mg-rich carbonate at low temperature does not follow the “classical nucleation theory”, but rather begins with the precipitation of an amorphous phase.^59–61^ The existence of a hypothetical precursor phase that is Mg-rich, amorphous, and forms exclusively under high saturation conditions is consistent with our observations. Our results show that precipitation of HMC is favored in the experiments whereby bicarbonate was mixed quickly with the solution (*i.e.*, BFM method). If the mixing is instead slow (*e.g.*, BSM method), the formation of other minerals such as aragonite reduces the saturation, which may in turn prevent the formation of the required precursor phase. Therefore, although the artificial solution is supersaturated with respect to dolomite already at pH 7 (but dolomite is not forming due to kinetic barriers^3^), it is possible that its hypothetical amorphous precursor requires a higher saturation. Such conditions should be reached fast enough to outcompete the formation of other phases. Among the methods tested in this study, only the BFM method would produce the mass-transfer rate necessary for the formation of the precursor phase. Future investigations including a continuous monitoring of the solid phases and high-resolution surface characterization of the precipitates may provide key insight to evaluate the hypothesis described above.

The influence of the precipitation method on the resulting mineral phase has been studied for calcium carbonate,^31^ showing that kinetics factors are important for determining polymorphism.^62^ In contrast, less is known on whether the dynamic of carbonate supply control the incorporation of Mg into carbonate minerals at low temperature. In 2012, Wang *et al.*,^60^ presented a conceptual model to explain the relationship between Mg^2+^ : Ca^2+^ ratio, carbonate supply and CaCO_3_ mineralization. They suggested that the combination “high Mg^2+^ : Ca^2+^ ratio” and “high saturation” favors the rapid formation of amorphous Mg-carbonates that eventually transforms into high Mg calcites. Although our results emphasize the importance of the “mode of carbonate supply” rather than the “carbonate ions concentration”, the outcome of our precipitation experiments is broadly consistent with their model. Indeed, from solutions characterized by an elevated Mg^2+^ : Ca^2+^ ratio, very high magnesium calcite (VHMC) formed exclusively with the BFM method (*i.e.*, with a fast supply of bicarbonates), whereas mostly aragonite formed by adding bicarbonate slowly (*i.e.*, BSM method). Nevertheless, also with a fast bicarbonate supply, it has not been possible to produce carbonate phases with a mol% Mg higher than 35. VHMC with a mol% Mg exceeding 40 was obtained exclusively in the presence of organic molecules. Because several factors other than Mg/Ca ratio are known to favor the incorporation of Mg in carbonate minerals (*e.g.*, temperature, presence of cell walls, extracellular polymeric substances, clay minerals, presence of some trace metals…) (*e.g.* ref. 15, 33, 34, 51, 63 and 64) it would be virtually impossible to produce a model that correctly predict mineral formation in different natural environments just as a function of velocity/mode of carbonate supply and Mg/Ca ratios.

Considering the results of our laboratory experiments, we hypothesize that also in natural sedimentary systems different carbonate minerals may form depending on the mode of carbonate supply. No values measured directly in the field are to our knowledge available for evaluating whether a carbonate supply analogue, for example, to that of the BMF method used in our experiments can be produced by a natural process. Nevertheless, we speculate that progressive evaporation of seawater may correspond to a carbonate supply that is not ideal for formation of Mg-rich carbonates, even in the presence of organic molecules. Instead, microbial metabolic reactions that releases at high-rate carbonate ions in pore-waters or within a microbial mat may result in a relatively faster carbonate supply, which is more similar to that obtained with the BFM method. For instance, the mode of carbonate supply could play an important role in freshwater–seawater mixing-zone, which have since long been studied to formulate models for low temperature dolomite formation.^2,65,66^ In mixing zones, not only there is a rapid mixing of solutions characterized by different chemical compositions, but conditions are particularly favorable for sustaining microbial metabolisms (*e.g.*, manganese-, iron-, and sulfate-reduction coupled to organic matter oxidation) that generates high rate of alkalinity in pore waters rich in organic molecules.^67^ Similarly, in supratidal organic-rich pore waters of modern sabkhas^30,50^ dolomite *vs.* aragonite formation may be determined not only by the presence of organic molecules (*i.e.*, living and degrading microbial cells and extracellular polymeric substances)^22^ but also by cyclic mixing of seawater brought by the tide with more evaporated seawater present in the sediment pores, in which the presence of hotspots of alkalinity production associated to microbial respiration of organic matter. Future studies measuring alkalinity production-rate and daily fluctuations in sabkhas, and other modern dolomite-forming environments will be essential to test the abovementioned hypothesis.

Regarding the morphologies of the precipitates, spherical shaped Mg–calcium carbonate crystals were observed in the ADFM precipitation experiments. Crystals with semi-spherical shapes were observed in the BSM experiments, while oval shaped crystals were obtained in the experiments performed at BFM method. These observations suggest that factors other than mol% Mg incorporated into the carbonate mineral are at play in determining crystal morphology (*e.g.* ref. 20). Thus, crystal morphology can hardly predict the composition of the carbonate nor the mechanisms through which it originally formed.

Each experiment was conducted at least in duplicate. In some cases, 3 or 4 parallel experiments were conducted. The error reported next to the pH and mol% Mg values refers to the differences among duplicates/parallel experiments.

## 4 Conclusions

The results of our laboratory precipitation experiments showed that, at equal physicochemical conditions, a fast supply of carbonate ions promote the incorporation of Mg into carbonate minerals at low temperatures. In an artificial solution that simulates evaporitic seawater (*i.e.*, sabkha water), Mg-carbonates with a mol% Mg of about 35 are formed exclusively when bicarbonate is mixed fast to the solution. If the solution also contains organic molecules, the mol% of Mg in the carbonate surpasses 40, forming a phase that is a possible precursor to ordered dolomite. These observations provide a possible explanation for why Mg-rich mineral phases rarely form in modern natural environments, despite the fact the microbes and extracellular polymeric substances are almost ubiquitous in all low temperature sedimentary systems: the mode of carbonate supply may be equally important as the presence of organic molecules. No correlation between the charge of the organic molecules and the mol% Mg of the carbonate minerals has been identified in our experiments. However, more tests covering a larger pH range are necessary to draw general conclusions on the importance of charged functional groups in natural environments. In future studies aimed at finding a definitive solution to the long-standing enigma that surrounds the incorporation of Mg into carbonate minerals at low temperature—leading to dolomite formation, it will be important to focus not only on biological factors, saturation indexes, and the total alkalinity of the system, but also on the dynamics controlling how carbonate ions are supplied to the site of mineral formation.

## Author contributions

ZA: conceptualized & designed the research, performed the experiments, analyzed the data, and wrote the manuscript. TB, NZ, HK, and FS: conceptualized & designed the research, analyzed the data, provided resources, equipment and infrastructure, and reviewed & edited the manuscript. MD: helped with conceptualization and formal analysis of the data, ES and MA helped with the experimental setup and data analysis.

## Conflicts of interest

The authors declare that this research was conducted in the absence of any commercial or financial relationships that could be construed as a potential conflict of interest. There are no conflicts to declare.

## Supplementary Material

RA-011-D1RA06206A-s001
