# Supplementary material for: Systematic laboratory approach to produce Mg-rich carbonates at low temperature
Source: RSC Adv. 2021 Nov 18;11(59):37029–39. doi: 10.1039/d1ra06206a (PMC9043586; doi:10.1039/d1ra06206a)
Supplement: RA-011-D1RA06206A-s001 [file RA-011-D1RA06206A-s001.pdf]

Electronic Supplementary Material (ESI) for RSC Advances.
This journal is © The Royal Society of Chemistry 2021

Systematic laboratory approach to produce protodolomite and Mg-rich carbonates at low temperature

Supplementary Data

1. Qatar Map

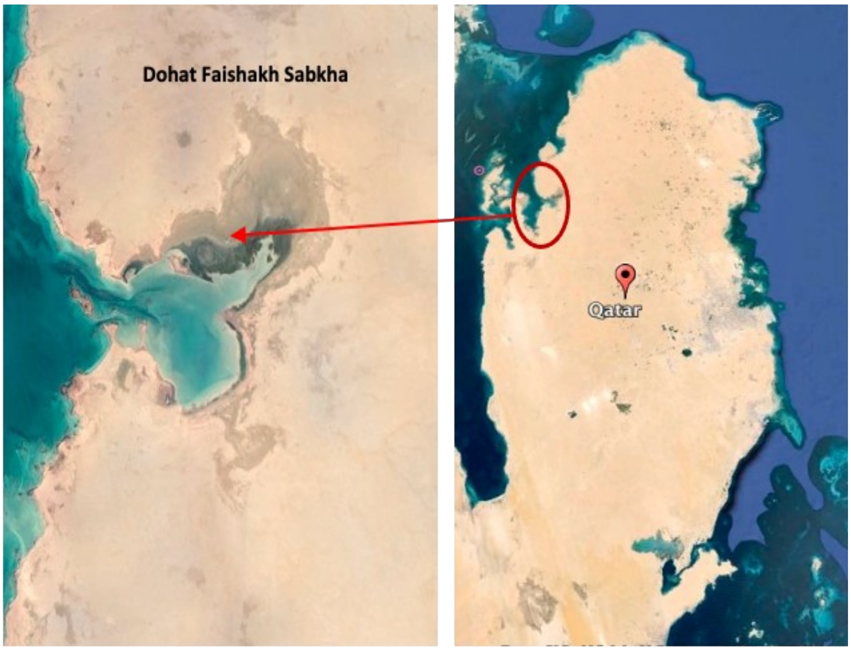

Supplementary Figure S1: Dohat Faishakh Sabkha located northwest of Qatar

2. Porewater sampling and analysis

Core samplers were prepared by crafting 5 cm apart holes along each core to collect porewater. The porewater was obtained from sediment cores by inserting Rhizon samplers from Rhizosphere into the holes, clean syringes were attached to extract the pore water. The collected pore water was transferred to sterile 50 ml centrifuge tubes for further analysis. Chemical analyses of the pore waters were performed by inductively coupled plasma (ICP)-mass spectroscopy was performed for Na^{+}, Mg^{2+}, K^{+} and Ca^{2+} cations, and Ion Chromatography (IC) for Cl^{-}, Br^{-}, Sr^{-} and $SO4^{-2}$ anions using the ICP-mass spectrometer Agilent 7500CX-United States as previously described[1]. The concentration of major ions in the porewater samples collected from samples core layers associated with occurrence of dolomite are shown in (Table S1).

Supplementary Table S1: Concentrations of major ions in the porewater samples collected from samples core layers associated with occurrence of dolomite.

Sample	pH	Na^{+} (mM)	Mg^{+2} (mM)	K^{+} (mM)	Ca^{+2} (mM)	Sr^{2+} (µM)	Li^{+} (µM)	Cl^{-} (mM)	$SO4^{-2}$ (mM)
PW1	7.15	3847	363	63	26	288	142	5221	106
PW2	7.17	4054	396	71	23	294	154	5209	103
PW3	7.17	4239	392	69	23	293	149	5231	105
PW4	7.07	4345	386	66	24	272	139	4878	101
PW5	7.02	4137	365	66	26	310	129	5151	95
PW6	7.23	4323	339	64	29	357	123	4878	90
Average	7.14	4158	374	66	25	302	139	5095	100

Pw: Porewater

3. Selected properties for the amino acids used in the study

Supplementary Table S2: Selected properties for the amino acids used in the study[2].

**Amino Acid**	**Abbreviation**	**Group of Amino Acid**	**pK_a**	**pK_b**	**pK_c Side Chain**	**Net charge at pH 7-8:30**
Alanine	Ala	Non-polar hydrophobic amino acids	2.34	9.69	---	Zwitterion form
Phenylalanine	Phe	Non-polar hydrophobic amino acids	1.83	9.13	---	Zwitterion form
Glutamine	Gln	Polar hydrophilic amino acids	2.17	9.13	---	Zwitterion form
Aspartic acid	Asp	Acidic amino acids	1.88	9.60	3.65	-1
Glutamic acid	Glu	Acidic amino acids	2.19	9.67	4.25	-1
Arginine	Arg	Basic amino acids	2.17	9.04	12.48	+1

pK_a , pK_b , pK_c , Negative of the logarithm of the acid dissociation constants for the COOH and NH_2 and side chain groups in the molecule (at 25 °C).

4. Saturation Indices

The Saturation Indices were calculated using the Geochemist's Workbench (GWB2021)-Community Edition.

Supplementary Table S3: Calculated Saturation Indices (Log Q/K) of Major Carbonate Minerals.

	Dolomite	Calcite	Aragonite	Monohydrocalcite	Magnesite	Huntite	Hydromagnesite	Nesquehonite
pH7	3.7539	0.5792	0.4151	-0.4743	1.5752	3.6096	-2.9536	-1.3562
pH8	5.3947	1.4297	1.2648	0.3895	1.9829	6.7056	1.9829	-0.5016
pH9	7.0834	2.2853	2.1204	2.1204	3.1693	10.607	7.2857	0.3315

Supplementary Table S4: Log Ion activity for Main Cations & Anions

**pH/Ion**	HCO_3^-	CO_3^{-2}	Mg^{+2}	Ca^{+2}
**pH7**	-1.64	-4.94	-1.49	-3.15
**pH 8**	-1.96	-4.3	-1.27	-2.9
**pH 9**	-2.1	-3.44	-1.3	-2.91

Supplementary Table S5: Ion Molar Concentrations and Ratios at different pH values.

	HCO_3^-	CO_3^{-2}	Ca^{+2}	Mg^{+2}	HCO_3^-/ CO_3^{-2}	Ca^{+2}/CO_3^{-2}	Mg^{+2} /CO_3^{-2}
pH7	0.03142	8.62E-05	0.003458	0.1088	364.50	40.12	1262.18
pH8	0.01468	0.0003685	0.005989	0.1751	39.84	16.25	475.17
pH9	0.01071	0.002688	0.005887	0.1635	3.98	2.19	60.83

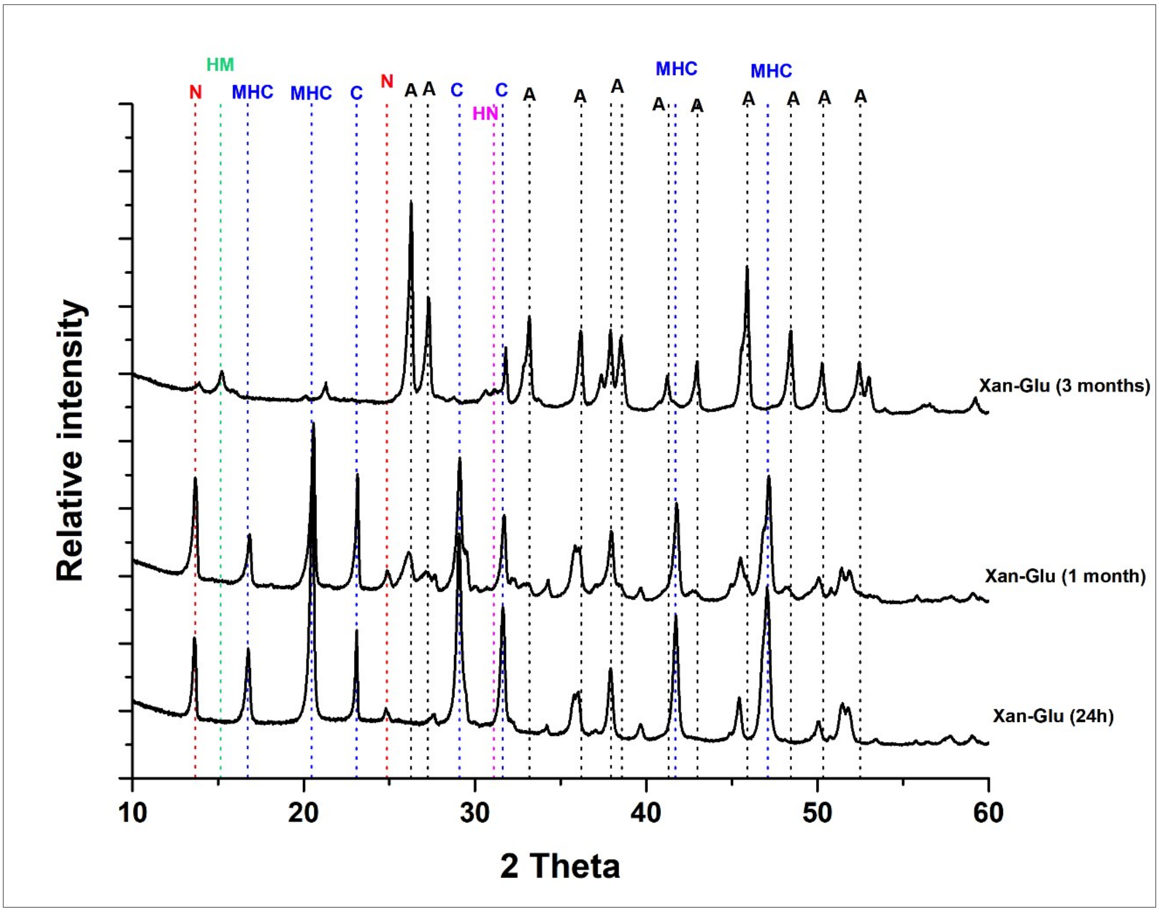

Supplementary Figure S2: Representative closeup of the XRD spectra showing transformation of minor phases. A: Aragonite, MHC: Monohydrocalcite, C: Calcite, N: Nesquehonite, HN: Huntite, HM: Hydromagnesite.

References:

1. N. Gros, "Ion Chromatographic Analyses of Sea Waters, Brines and Related Samples," *Water,* vol. 5, no. 2, pp. 659-676, 2013.
2. D. R. Lide, CRC Handbook of Chemistry and Physics, 2003-2004.
